# Warfarin dose and INR related to genotypes of CYP2C9 and VKORC1 in patients with myocardial infarction

**DOI:** 10.1186/1477-9560-6-7

**Published:** 2008-06-17

**Authors:** Kari Bente Foss Haug, Mohammad N Sharikabad, Marianne K Kringen, Sigrid Narum, Stine T Sjaatil, Per Wiik Johansen, Peter Kierulf, Ingebjørg Seljeflot, Harald Arnesen, Odd Brørs

**Affiliations:** 1 R&D, Department of Clinical Chemistry, Ulleval University Hospital, Kirkeveien 166, O407 Oslo, Norway; 2 Center for Clinical Research, Ulleval University Hospital, Kirkeveien 166, 0407 Oslo, Norway

## Abstract

**Background:**

Warfarin treatment has a narrow therapeutic range, requiring meticulous monitoring and dosage titration. Individual dosage requirement has recently partly been explained by genetic variation of the warfarin metabolizing enzyme CYP2C9 and the Vitamin K-activating enzyme VKORC1. In the WARIS-II study, comparing three different antithrombotic regimens after myocardial infarction, warfarin treatment reduced thrombotic events, but was associated with more frequent bleeding than use of acetylsalisylic acid (ASA) alone.

**Aims:**

The primary aim of the present study was to investigate the relation between genotypes of CYP2C9 and VKORC1 and warfarin maintenance dose in myocardial infarction. The secondary aim was to relate the genotypes to international normalized ratio (INR).

**Methods:**

Genotyping was performed in 212 myocardial infarction patients from the WARIS-II study by robotic isolation of DNA from EDTA whole blood (MagNa Pure LC) before PCR amplification (LightCycler) and melting point analysis.

**Results:**

The 420 C>T substitution of CYP2C9*2, the 1075 A>C substitution of CYP2C9*3 and the 1173 C>T substitution of VKORC1 had minor allele frequencies of, 11.3%, 5.7% and 36.6% respectively. Warfarin weekly dose varied between 17 mg and 74 mg among the patients. INR did not vary between genotypes. Warfarin dosage requirement was significantly associated with CYP2C9 and VKORC1 genotypes, treatment group and age. The VKORC1 genotype contributed 24.5% to the interindividual variation in warfarin dosage, whereas the combined CYP2C9 genotypes were only responsible for 7.2% of the dose variation.

**Conclusion:**

CYP2C9 and VKORC1 genotype frequencies in myocardial infarction patients appear similar to other patient groups and have similar impact on warfarin maintenance dose.

## Background

Warfarin and aspirin (ASA) have a well established role in secondary prevention of atherothrombotic disease, reducing new thromboembolic events [1-4]. However, response to anticoagulant treatment varies between individuals, requiring careful monitoring in order to keep international normalized ratio (INR) within a narrow therapeutic range. In spite of adherence to dosage regimens, INR values have been observed to be outside the target range 50% of the time [5,6], and this could possibly lead to treatment failure or adverse events. An important issue is to improve anticoagulation treatment in order to avoid thrombosis and treatment-induced bleeding.

Warfarin antagonizes the vitamin K-dependent activation of a range of coagulation factors (II, VII, IX, X) and anticoagulants (protein C, protein S), and INR is used as an indicator of coagulation status. Two gene products known to influence warfarin dose are the enzymes Cytochrom P 450 subtype 2C9 (CYP2C9) and the Vitamin K Epoxide Reductase 1 (VKORC1), which are involved in drug metabolism and vitamin K activation, respectively. Common gene polymorphisms exist for both enzymes, resulting in marked alteration of enzyme activity, and several studies have characterized the role of these polymorphisms in explaining a substantial part of the variation in warfarin dosage requirement [7-15]. In the study of Aithal *et al*. [16], carriers of CYP2C9 gene polymorphisms were affected by bleeding complicatins more often than non-carriers during warfarin treatment.

In the WARIS-II study, warfarin alone or in combination with low dose ASA (75 mg daily) were superior to 160 mg ASA in prevention of new thrombotic events after acute myocardial infarction, but was also associated with higher risk of bleeding [17]. Thus, 15.0 – 16.7% of the patients in the warfarin groups experienced the primary endpoint (new thrombotic events or fatal bleeding) and 11.3–13.1% experienced minor or major nonfatal bleeding during four years treatment. In comparison, ASA alone resulted in new thrombotic events in 20% of the patients and minor or major nonfatal bleeding in 4.0%. It is not known whether different frequencies of gene polymorphisms in the treatment groups contributed to the differences in bleeding risk.

The primary aim of the present study was to investigate the relation between genotypes of CYP2C9 and VKORC1 and warfarin maintenance dose in myocardial infarction patients (from the WARIS-II study). The secondary aim was to relate the genotypes to international normalized ratio (INR).

## Methods

### Patients

This substudy was established from the Warfarin Aspirin Reinfarction Study (WARIS-II), a Norwegian multicenter study, comparing three different antithrombotic regimens on clinical end-points of mortality, reinfarction and cerebral stroke after acute myocardial infarction [17]. All patients provided written informed consent before participation in the study. Three groups of patients were randomly assigned to treatment with either a daily dose of 160 mg ASA (Albyl E, Nycomed Pharma, Norway), warfarin (Marevan, Nycomed Pharma) with a target international normalized ratio (INR) of 2.8 to 4.2, or 75 mg of ASA combined with warfarin (target INR 2.0 – 2.5) and followed for 4 years. Coagulation status of the warfarin patients was controlled by recording INR systematically. The present population consisted of totally 212 patients from the Oslo subset of the study at Ullevaal University Hospital, from whom we acquired blood samples for genotyping. In 105 of these, INR values and weekly warfarin dose were also available (58 in the warfarin group (W), and 47 in the W+ASA group). All bleeding episodes were registered throughout the study period [18].

### Genotyping

DNA was isolated from 200 μl EDTA whole blood by MagNA Pure LC robot [19] according to the manufacturer's instructions using MagNA Pure LC DNA High Performance isolations kit (Roche cat no 03003990001). DNA was amplified by polymerase chain reaction (PCR) on a real-time fluorescence LightCycler instrument [19] in a final volume of 20 μl using a LightCycler Faststart DNA Master Hybridization Probe Kit (Roche cat no 12239272001) with primers and probes specific for selected single nucleotide polymorphisms (SNPs) localized in the CYP2C9 and VKORC1 genes. Genotypes were determined by melting point analyses. PCR conditions were essentially containing FastStart Reaction Mix including FastStart Taq Polymerase, 3 mM MgCl_2 _and 50–100 ng genomic DNA.

Genotyping the two functional sequence variants CYP2C9* 2 and CYP2C9*3 in the human CYP2C9 gene (GenBank accession number AY702706) was performed essentially as described by others [20], except for the use of double probe concentration (0.4 μM) in the CYP2C9*2-assay. The CYP2C9*2 allele is a C to T substitution in nucleotide position 420 in exon 3, leading to an amino acid shift from arginine to cysteine in position 144. The CYP2C9*3 variant is an A to C shift in nucleotide position 1075 in exon 7, resulting in an amino acid change from isoleucine to leucine in position 359.

From common and publicly available sequence variants in the human VKORC1 gene (GenBank accession number AY587020), we selected one SNP (rs9934438/1173 C>T) localized in the first intron of VKORC1, reported to have a minor allele frequency (MAP) of about 40% [8] and to be in strong linkage disequilibrium with three other SNPs (rs9923231/17878363, rs8050894 and rs2359612). Patients carrying the rs9934438 variant have been shown to require less warfarin dosages [14] and Geisen *et al*. [15] suggested these four SNPs to be combined in a common VKORC1*2 haplotype, corresponding as a marker for low warfarin requirement. The forward and reverse primers were 5'-AAAAGCAGGGCCTACG-3' and 5'-CCGAGAAAGGTGATTTCCA-3', respectively. LCRed640-labelled sensor probe was 5'-CGACCCTTGGACTAGGATGG-3', whereas the anchor probe was 5'-GCCCGGTGCCAGGAGATC-3' [21]. The primer concentration was 0.5 μM, whereas the anchor and sensor probe concentrations were 0.4 μM and 0.2 μM, respectively. The following cycle conditions were used: 10 min denaturation at 95°C before 35 cycles at 95°C for 10 s, 60°C for 10 s and 70°C for 5 s. Melting conditions were 95°C for 0 s, 45°C for 30 s and temperature transition from 45°C to 85°C (0.1°C/s).

PCR primer and probe sequences were tested for homology with other sequences at the NCBI gene BLAST website [22]. All assays were validated by sequencing the PCR fragments for the different SNPs before genotyping the patients. Positive mutation controls and negative controls were included in every run.

### Statistical analysis

Statistical analysis was performed using SPSS 14.0 statistic software (SPSS Inc., Chicago, IL, USA). Univariate and stepwise multiple regression analyses were performed to evaluate the impact of polymorphisms in VKORC1 and CYP2C9 genes on mean warfarin dose, after adjustment for the covariates age, sex and treatment group. The significance level of *P *≤ 0.05 was set for entry into the model and P ≥ 0.10 for removal from the model.

## Results

### Patient characteristics

A total of 212 Caucasian patients, divided into three different anticoagulation regimens (see Materials and Methods) were genotyped for common VKORC1 and CYP2C9 polymorphisms in this study. One hundred and five patients were included in the two warfarin groups. The treatment groups were similar as to baseline characteristics (age, sex). Noteworthy, there was a statistically significant (P = 0.005, Mann-Whitney test) difference in mean warfarin dose between the warfarin and warfarin +ASA groups (41.7 mg/week vs. 34.4 mg/week, respectively). This difference reflects the differences in observed INR values 2.4–3.6 (mean 3.1) and 1.9–2.9 (mean 2.2) for warfarin and warfarin +ASA groups, respectively and in INR target values of 2.8–4.2 and 2.0–2.5, respectively.

### CYP2C9 and VKORC1 genotypes

Genotype distributions of CYP2C9*2, CYP2C9*3 and VKORC1 1173 C>T polymorphisms in 212 patients from the Oslo subset of the WARIS-II study are summarized in Table 1. The frequencies of homozygotes for the *2 and *3 CYP2C9-variants were as expected low (1.9 and 0.5%, respectively), whereas homozygotes of VKORC1 1173 C>T (T/T) was 13.2%. The frequencies of the two heterozygous genotypes CYP2C9*1/*2 and CYP2C9*1/*3 were 17.9% and 9.4%, respectively, whereas compound heterozygotes of CYP2C9*2/*3 was 0.9%. VKORC1 1173 C>T heterozygotes was 46.7%. Minor allele frequencies (MAF) of the CYP2C9*2 and CYP2C9*3-variants were 11.3 and 5.7%, respectively, and of the VKORC1 36.6%. The genotype frequency of the CYP2C9*1/*2 variant was 20.7% in the warfarin group and 17.0% in the warfarin + ASA group, whereas the CYP2C9*1/*3 variant was 8.6% in the warfarin group and 4.3% in the warfarin + ASA group. VKORC1 heterozygote genotype frequency was 50.0% in the warfarin group and 36.2% in the warfarin+ASA group. Homozygotes of the CYP2C9*2 variant were not present in the warfarin group, but constituted 8.5% of the warfarin + ASA group. CYP2C9 compound heterozygotes (CYP2C9*2/*3) was 1.7% in the warfarin group, but absent in the warfarin + ASA group. MAF frequencies for the *2 og *3 variants of CYP2C9 and VKORC1 in the warfarin group were 11.2, 5.2 and 40.5%, respectively, and 17.0, 2.1 and 33.0% for the respective variants in the warfarin + ASA group.

**Table 1:** Genotype distributions of VKORC1 1173 C>T, CYP2C9*2 and CYP2C9*3 polymorphisms in the investigated patients (MAF = minor allele frequency).

**Genotype**	**Total material N = 212**		**Warfarin N = 58**		**ASA N = 72**		**Warfarin + ASA N = 47**	
	
	**Number of patients (genotype frequency in %)**	**MAF (%)**	**Number of patients (genotype frequency in %)**	**MAF (%)**	**Number of patients (genotype frequency in %)**	**MAF (%)**	**Number of Patients (genotype Frequency in %)**	**MAF (%)**
**VKORC1**								
1173 C>T		36.6		40.5		36.1		33.0
CC	85 (40.1)		20 (34.5)		30 (41.7)		23 (48.9)	
CT	99 (46.7)		29 (50.0)		32 (44.4)		17 (36.2)	
TT	28 (13.2)		9 (15.5)		10 (13.9)		7 (14.9)	
**CYP2C9**								
*1/*1	147 (69.3)		40 (69.0)		48 (66.7)		33 (70.2)	
*1/*2	38 (17.9)	11.3	12 (20.7)	11.2	10 (13.9)	7.6	8 (17.0)	17.0
*2/*2	4 (1.9)		-		-		4 (8.5)	
*1/*3	20 (9.4)	5.7	5 (8.6)	5.2	12 (16.7)	10.4	2 (4.3)	2.1
*3/*3	1 (0.5)		-		1 (1.4)		-	
*2/*3	2 (0.9)		1 (1.7)		1 (1.4)		-	

### Associations of CYP2C9 and VKORC1 polymorphisms with warfarin dose and INR

**Figure 1:**
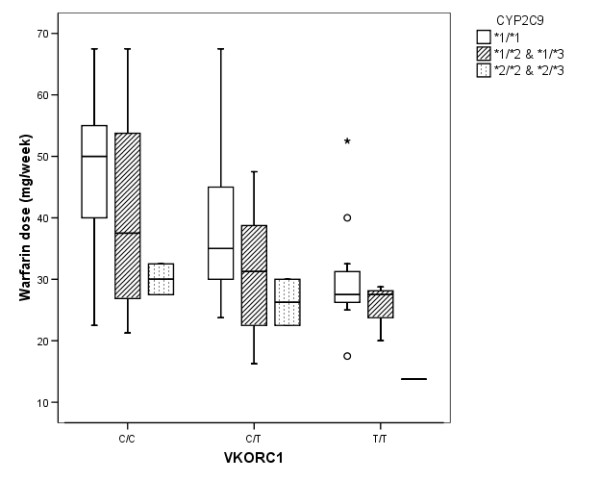
Box plot of mean weekly warfarin doses for different genotypes of VKORC1 1173 C>T and CYP2C9 *1,*2 and *3. The box represents the values from the 25 to 75% percentile. The middle line represents the median. The vertical line extends from the minimum to the maximum value, excluding outlier and extreme values which are marked as open circles and an asterisk respectively.

Relation between warfarin dose and CYP2C9 and VKORC1 genotypes is illustrated in Figure 1. The investigated polymorphisms in CYP2C9 or VKORC1 were associated with marked reduction in warfarin dosage requirements. Carriers of CYP2C9 polymorphisms with wildtype VKORC1 genotype (C/C) show almost similar reduction in dose requirement as carriers of VKORC1 polymorphism with wildtype CYP2C9 (*1/*1). Results of the stepwise multivariate regression analysis of the two warfarin groups (totally 105 patients; 58 warfarin; 47 warfarin + ASA) are given in Table 2. All three polymorphisms of CYP2C9 and VKORC1 covaried significantly with warfarin dose according to the regression model. The investigated VKORC1 polymorphism showed closest association with the variation in warfarin dose, explaining 24.5% of the interindividual variability, whereas the two CYP2C9 polymorphisms in combination contributed to 7.2% of the dose variation. Age accounted for 4.2% of the total dose variability, whereas sex did not contribute to dose variation.

**Table 2:** Multiple regression analysis of mean weekly warfarin dose (mg) related to genotypes, treatment group and age. Coefficients and significance levels are listed for each predictor. R^2 ^values indicate the cumulative contribution of each predictor as they are entered into the model. P-value for the model was <0.0001.

**Predictor**	**Warfarin dose (mg)**	**Significance**	**Adjusted R^**2 **^(cumulative)**
Constant	72.297	P < 0.001	
VKORC1 1173 T/T	-18.946	P < 0.001	**0.101**
VKORC1 1173 C/T	-11.930	P < 0.001	**0.245**
Treatment group	-8.540	P < 0.001	**0.358**
CYP2C9 *1/*2,*3	-7.275	P = 0.001	**0.398**
CYP2C9 *2/*2,*3	-15.459	P = 0.001	**0.430**
Age	-0.321	P = 0.004	**0.472**

Dose [mg/week] = 72.297 - 18.946 · (T/T) -11.930· (C/T) - 8.540 · (group) -7.275 · (*1/*2,*3) - 15.459 · (*2/*2,*3) - 0.321· (age)

INR values did not vary significantly between different genotypes as shown in Table 3. There were observed 2 major, 7 minor and 14 occult bleeding episodes in the present material of 105 warfarin-treated patients. As shown in Table 3, there is a trend for more bleeding episodes in patients with the C/T VKORC1 genotype. There were 2 major, 6 minor and 7 occult bleeding episodes in the warfarin group in comparison with one minor and 7 occult bleeding episodes in the warfarin + ASA group, a difference that could be related to higher INR (0.9 unit) in the warfarin group.

**Table 3:** Bleeding events according to CYP2C9 and VKORC1 genotype.

**CYP2C9 genotype**	**VKORC1 genotype**	**N (%)**	**Mean INR (95% C.I.)**	**Major/minor bleeding events N (%)**	**Occult bleeding events N (%)**	**Total bleeding events N (%)**
*1/*1	C/C	33 (31.4)	2.65 (2.45–2.84)	2 (6.1)	4 (12.1)	6 (18.2)
	C/T	28 (26.7)	2.79 (2.60–2.99)	3 (10.7)	5 (17.9)	8 (28.6)
	T/T	12 (11.4)	2.71 (2.37–3.05)	2 (16.7)	-	2 (16.7)
*1/*2,*3	C/C	8 (7.6)	2.58 (2.19–2.96)	-	1 (12.5)	1 (12.5)
	C/T	16 (15.2)	2.83 (2.55–3.10)	2 (12.5)	4 (25.0)	6 (37.5)
	T/T	3 (2.9)	2.87 (2.49–3.25)			
*2/*2,*3	C/C	2 (1.9)	2.30 (1.03–3.57)			
	C/T	2 (1.9)	2.15 (0.24–4.06)			
	T/T	1 (1.0)	3.50			

**Total**		105 (100)		9	14	23

## Discussion

This study was designed to investigate the frequency of CYP2C9 and VKORC1 gene polymorphisms and their relation to warfarin dose and INR in patients with myocardial infarction from the Norwegian WARIS-II study. The genotype and allele frequencies of CYP2C9 and VKORC1 polymorphisms for the whole group of 212 patients investigated were in accordance with recent reports from studies in Caucasians. The relation observed between polymorphisms of CYP2C9 and VKORC1 and variation in warfarin dose requirement was as previously reported in other patient groups.

The minor allele frequencies (MAF) of 37% for the VKORC1 polymorphism 1173C>T, 11% for the CYP2C9*2 and 6% for the CYP2C9*3 were all slightly lower than those reported by Takahashi, who found MAF of 42, 14 and 10% for the respective polymorphisms in Caucasians [9]. The results from the multivariate analysis of the present 105 Norwegian patients of Caucasian race confirmed previous studies with regard to apparent influence of the genotypes on warfarin dosage requirement, the VKORC1 polymorphism having the larger impact (25%) compared to CYP2C9 (7%). In a group of 201 Swedish patients, VKORC1 contributed 29% and CYP2C9 12% [8]. Another study performed with 165 Slovenians showed that VKORC1 contributed 34% and CYP2C9 18% [23]. As recently reviewed by Wadelius and Pirmohamed [24], six studies found a greater relative contribution of VKORC1 than CYP2C9 [8-10,12,13,15]; two studies found that CYP2C9 had a greater contribution [11,14], and one study found equal contribution from the two polymorphisms [7]. The relative contribution of each polymorphism to variation in warfarin dosage requirement in the whole group of patients must be considered in relation to the frequencies of the polymorphisms. Consequently, VKORC1 likely contributes more than CYP2C9 due to markedly higher frequency of the 1173C>T polymorphism than of the CYP2C9*2 and CYP2C9*3 polymorphisms. However, in the individual patient, the CYP2C9*2 and especially the CYP2C9*3 polymorphism will be expected to influence warfarin dosage requirement markedly [25]. This is in accordance with our data, showing equal impact on warfarin dosage variation from polymorphisms in VKORC1 and CYP2C9 when investigated separately (Figure 1).

A meta-analysis of 33 studies showed that major and fatal bleedings occurred at rates of 7.2 and 1.3, respectively, per 100 patients years during oral anticoagulant therapy [26]. Aithal *et al*. [16] found that carriers of CYP2C9 polymorphisms had higher occurrence of bleeding than non-carriers during warfarin treatment. Carriers of alleles coding for reduced CYP2C9 and/or VKORC1 enzyme activity, requiring lower warfarin doses, have been observed to be more difficult to titrate to a stable maintenance dose than those needing larger doses [27]. A meta-analysis of CYP2C9 genetic polymorphisms showed that the relative bleeding risk was 1.91 for CYP2C9*2 and 1.77 for CYP2C9*3. For either variant, the relative risk was 2.26 [28]. This suggests that polymorphism carriers have a predisposition for bleeding complications during therapy [29]. When considering the occurrence of bleeding episodes in different genotypes (Table 3), it is obvious that this study is underpowered to detect differences in bleeding risk. Nevertheless, it appears to be more bleeding episodes in patients with the C/T VKORC1 genotype as shown in Table 3.

The possible impact of the warfarin dose-related polymorphisms on risk of myocardial infarction has recently been studied. Yasar *et al*. found a higher risk of myocardial infarction in individuals carrying the *2 or *3 variants of CYP2C9 [30], whereas Funk *et al*. [31] found that the *2 and *3 allele variants of CYP2C9 were associated with a lower risk of myocardial infarction in males (Odds Ratio 0.56), but not in females. Wang *et al*. [32] found a significant association between the rs2359612/2255 C>T VKORC1 polymorphism (a polymorphism found to be in strong linkage disequilibrium with rs9934438/1173 C>T) and the risk of coronary heart disease (Odds Ratio for C carriers 1.72). Obviously, more studies are needed to investigate the relationship between genotypes, risk of thromboembolic events (coronary heart disease and myocardial infarction) and treatment-induced bleeding.

In conclusion, the observed genotype frequencies of CYP2C9 and VKORC1 in patients with myocardial infarction are similar to those previously reported in other patient groups and healthy subjects in Caucasians, as is the relation between warfarin dosage and genotypes. Genotyping would be of value for secondary stratification of patients into comparable study groups when testing different treatment regimens. The relation between gene polymorphisms and bleeding frequency during warfarin treatment needs to be further investigated in larger studies with sufficient statistical power.

## Abbreviations

ASA: aspirin; CYP: cytochrom P 450; INR: international normalized ratio; MAF: minor allele frequencies; SNP: single nucleotide polymorphism; VKORC1: vitamin K epoxide reductase 1; WARIS-II: Warfarin Aspirin Reinfarction Study.

## Competing interests

The authors declare that they have no competing interests.

## Authors' contributions

KBFH participated in the study design, developed and followed up the genetic analysis and drafted the manuscript

MNS participated in the study design, performed the statistical analysis and drafted the manuscript

MKK participated in the study design, performed the statistical analysis and drafted the manuscript

SN participated in the study design

STS performed the genotyping

PWJ participated in the study design and drafted the manuscript

PK participated in the study design and drafted the manuscript

IS contributed with the WARIS-II material

HA contributed with the the WARIS-II material

OB participated in the study design and drafted the manuscript

All authors read and approved the final manuscript.
